# Hypofertility in Muckle Wells syndrome and treatment with IL-1 targeting drugs

**DOI:** 10.1186/1546-0096-9-S1-P14

**Published:** 2011-09-14

**Authors:** Ulrich Meinzer, Isabelle Marie, Isabelle Koné-Paut, Tu-Anh Tran

**Affiliations:** 1 Division of Paediatric Rheumatology and Division of Paediatrics, CEREMAI, Hôpital de Bicêtre; University of Paris Sud, Le Kremlin Bicêtre Cedex, France

## Introduction

Muckle-Wells syndrome (MWS) is an inherited autoinflammatory disease (SAID) caused by NLRP3 gene mutations, which results in excessive interleukin-1b (IL-1) secretion. MWS is characterized by recurrent bouts of systemic inflammation, resulting in fever, rash and joint pain. Main complications include sensorineural deafness and amyloidosis. Infertility was mentioned in the first description of MWS but this complication has never been thoroughly studied. Interleukin-1 targeting drugs were shown to alleviate the clinical symptoms of MWS patients and to improve their quality of life, but nothing is known on its potential ability to reverse infertility.

## Aims

To assess the fertility of male patients with MWS and to analyse the effect of anti-IL1 on male MWS infertility

## Methods

Medical records of male MWS patients followed in our tertiary center for SAID were reviewed retrospectively for data indicating fertility problems. When patients agreed spermiograms were performed.

## Results

Seven patients (17y, 21y, 31y, 43y, 45y, 46y, 47y) were identified. All carried R260W NLRP3 mutations and presented typical clinical and biological signs of MWS. None had amyloïdosis or risk factors for infertility. Four patients having regular sexual activity were unable to have children after 2 years; two used in vitro fertilisation with donor sperms. Spermiograms (available in 5 patients) showed azoospermia (3 patients) and oligospermia (2 patients). In three patients spermiograms were available before and after treatment with canakinumab. Two patient had been treated with anakinra, an IL-1 receptor antagonist, during 2 years, prior to be switched to canakinumab. All three patients received canakinumab during 2-4 years. Canakinumab induced clinical and biological remission but did not change azoospermia or oligospermia.

## Conclusions

Male MWS patients may be sterile. Better knowledge on the natural history of azoospermia would help to discriminate the respective role of NLRP3 mutation, chronic inflammation, and other factors. Late intervention by IL-1 blockade treatment seems to be ineffective. Additional studies are required to better establish the frequency of infertility and to investigate whether early treatment with interleukin-1 targeting drugs before puberty may prevent sterility.

